# Optimizing the Care and Health of Women with Inflammatory Bowel Disease

**DOI:** 10.1155/2015/435820

**Published:** 2015-05-21

**Authors:** Judy Nee, Joseph D. Feuerstein

**Affiliations:** Department of Medicine and Division of Gastroenterology, Beth Israel Deaconess Medical Center, Harvard Medical School, 8E Gastroenterology, 110 Francis Street, Boston, MA 02215, USA

## Abstract

Inflammatory bowel disease (IBD) including both ulcerative colitis and Crohn's disease is increasing worldwide. Although diagnosis is equally found in men and women, the chronicity of IBD poses a unique impact on the milestones of a woman's life. As the gastroenterologist becomes increasingly important in the health maintenance of patients with IBD, this review stresses the unique gender issues in women with IBD related to menstruation, cervical cancer, sexual health, contraception, and menopause that may affect the course of disease, treatment decisions, and quality of life.

## 1. Introduction

Inflammatory bowel disease (IBD) is a chronic idiopathic inflammatory bowel disease which includes both ulcerative colitis (UC) and Crohn's disease (CD) [1, 2]. The two diseases are both inflammatory conditions but present very differently and involve different parts of the gastrointestinal tract. UC was first described by Wilks in the 1800s [3]. It is an inflammatory disease that is characterized by continuous inflammation of the colonic mucosa that extends proximally from the rectum [1]. The natural history of the disease ranges from periods of quiescent remission to flares. The mainstay of treatment is pharmacologic therapy. However, up to 30% of individuals will necessitate total proctocolectomy due to refractory disease, dysplasia, or the development of cancer [4, 5]. The standard surgical options include total proctocolectomy and end-ileostomy, proctocolectomy with ileoanal pouch anastomosis (IPAA), and, less frequently recommended, abdominal colectomy with ileorectal anastomosis [4]. Crohn's disease was first reported by Crohn et al. in 1932 [6]. It can involve any portion of the gastrointestinal tract from mouth to anus. It is classically characterized by skip lesions in which there are discrete areas of diseased bowel separated by normal bowel and typically it spares the rectum [2]. Approximately 50% of patients have their disease involving the terminal ileum and colon, 30% have isolated small bowel disease, 20% have isolated colonic disease, and up to 25% will also have perianal complications [2]. Crohn's disease can present in many different ways often based on the underlying disease phenotype. The three classic types of Crohn's disease include inflammatory disease (nonpenetrating and nonstricturing), stricturing disease, and fistulizing disease. Similar to UC, the natural history of disease varies between remission and flares. The mainstay of therapy is pharmacologic management of disease [2]. However, surgery will be necessary in up to 80% of individuals with 50% undergoing a surgery within 10 years from their diagnosis [5, 7]. Unlike UC, the surgical management of Crohn's disease is not curative. Therefore, the types of surgery are dictated by the presenting problem (e.g., stricture or fistula) and a goal of removing the smallest amount of bowel necessary.

Both CD and UC are increasing in prevalence globally with as many as 1.4 million individuals in the United States and 2.2 million individuals throughout Europe [8, 9]. The overall incidence of IBD in pediatrics is rising worldwide. Benchimol et al. reviewed 139 studies from 32 different countries from 1950 to 2009 to assess the worldwide epidemiology of childhood-onset IBD. They noted that, in studies reporting statistical trends, there has been a 60% increase in childhood onset Crohn's disease and 20% increase in UC [10]. Similarly, the incidence of IBD in adults is also rising globally with the highest prevalence in Europe and North America [11]. The peak incidence of IBD is between 15 and 30 years old [12]. IBD affects men and women equally. An important difference between pediatric onset and adult onset IBD is that pediatric onset IBD is associated with more extensive intestinal involvement and a more rapid progression of their disease [13, 14].

However, because IBD is a chronic illness, the impact of IBD on women is unique. IBD may affect the major milestones of a woman's life including menstruation, sexuality, family planning, and menopause. Rarely discussed topics such as how medications and surgery may adversely affect self-image are critical to broach with patient. As the gastroenterologist becomes increasingly important in the health maintenance of patients with IBD, this review seeks to stress the unique gender issues in women with IBD related to menstruation, cervical cancer, sexual health, contraception, and menopause that may affect the course of disease, treatment decisions, and the quality of life.

## 2. Body

### 2.1. Quality of Life

The overall reported quality of life (QOL) with IBD is variable and is often dependent on symptom severity [15, 16]. Often, CD is reported to have a greater negative impact on QOL compared to UC [16]. One challenge affecting the overall QOL is that typically individuals are not comfortable discussing their gastrointestinal symptoms in an open forum with friends and colleagues [16]. In a survey of 5,576 patients, 75% of patients reported that their symptoms affected their ability to enjoy leisurely activities and 69% reported that their symptoms affected their ability to perform at work [15]. Importantly, though, over 70% of individuals who were on therapeutic agents reported improvements in their overall QOL [15]. Similarly, while the QOL with severe uncontrolled UC or immediately postsurgery was suboptimal, improvement in QOL was already noted 1 month after ileostomy take-down and returned to normal within one year after surgery for UC [17]. Potential predictors of reduced QOL include those with a higher perceived level of stress, less social support, greater number of disease relapses, and female sex [18].

When therapeutic modalities are successful in putting the disease in remission QOL is usually similar to the general population. However, when symptoms are not well controlled, especially in pediatrics, there can be a significant reduction in QOL [19]. Additionally, when therapeutic options result in side effects this further diminishes one's perception of QOL [20]. Importantly, the reduction in overall QOL in adolescence with IBD may be present regardless of disease activity. A study by MacKner et al. reported that patients with IBD had poorer school functioning compared to age matched controls [21].

Throughout this review the effects of women's issues in IBD and how they affect QOL will be addressed.

### 2.2. Menstruation

#### 2.2.1. Puberty and Menarche

IBD may delay the onset of menses in adolescence, especially when the disease is poorly controlled [22, 23]. The exact etiology for this delay is unclear. Possible causes for this delay include growth failure from being underweight and nutritional deficits [22]. Medication usage, as the case with corticosteroids, also contributes to growth retardation and delayed puberty [24]. Additionally, in animal models, inflammatory mediators in active colitis may also contribute to malnourishment and pubertal delay. However, once disease is in a sustained remission, puberty and menarche occur soon after [25].

#### 2.2.2. Symptoms and Changes during Menstrual Cycle

The hormonal changes during the menstrual cycle may affect a multitude of chronic disease symptoms and IBD is no exception [26]. In a study of 238 patients with IBD (151 CD, 87 UC) and 156 healthy controls, patients with CD were more likely to complain of an increase in diarrhea prior to and during menses, whereas patients with UC complained of an increase in diarrhea only during menses [27]. A recent study by Saha et al. found that changes in menses occurred even prior to the diagnosis of IBD [28]. In the year prior to the diagnosis of IBD, 21% of patients developed a change in the duration of menstrual flow [28]. Additionally, those suffering from dysmenorrhea had an increase in intensity of menstrual pain. This resulted in a significantly lower quality of life compared to those with more regular menstrual cycles. Over time, though, cycles became more regular [28].

If the physician and/or patients are not aware of these expected changes, many of them may be misinterpreted as exacerbations of the underlying IBD [29]. Therefore, counseling patients about expected fluctuations in symptoms that occur during the menstrual cycle is important in improving their overall quality of life.

### 2.3. Cervical Cancer Prevention and Screening

#### 2.3.1. Risk of Cervical Cancer

Kane et al. reported that the overall incidence of any abnormal pap smear in a woman with IBD was 42.5% compared to 7% of controls [30]. Furthermore, women with IBD were more likely to be diagnosed with higher-grade lesions than controls [30]. Though not statistically significant, there was a trend toward those on immunosuppressants for greater than 6 months to have more abnormal pap smears [30]. The data on whether or not thiopurines increase the risk of cervical dysplasia is equivocal [31–33]. Recently, however, another population based study raises the possibility that IBD alone, regardless of thiopurine usage, may increase the risk of cervical neoplasia [34].

#### 2.3.2. Screening for Cervical Cancer

Currently, the cervical cancer screening is recommended in the general female population every 2-3 years [35]. However, immunosuppressed patients, such as those with HIV, have recommendations to undergo yearly pap smears [36]. Given the equivocal evidence regarding the risk of cervical dysplasia from thiopurine usage, some practices, like ours, recommend yearly pap smears. Nevertheless, yearly pap smears have not been advocated by major societies. Despite this concern for cervical dysplasia in this population, Singh et al. found that just over 50% of women with IBD underwent pap smears at guideline recommended intervals. Independent predictors of lower adherence to pap smears were CD and immunosuppressant usage [37].

#### 2.3.3. Prevention of Cervical Cancer

Human papilloma virus (HPV) vaccinations, such as Cervarix and Gardasil, target HPV types 16 and 18, which are responsible for 70% of cervical cancers. Currently the Advisory Committee on Immunizations Practices (ACIP) recommends vaccinations for girls 9 years to 26 years of age [38]. Given the risk of abnormal pap smears, women on chronic immunosuppression are also candidates for HPV vaccination regardless of sexual activity status. Despite the beneficial effects of HPV vaccination, Wasan et al. found that the overall knowledge of gastroenterologists regarding appropriate vaccinations is poor [39].

### 2.4. Sexual Health

#### 2.4.1. General Issues in Sexual Health

IBD can have a significant impact on a woman's sexual well-being [40]. Sexual activity may be a major component in considering what is “healthy.” Sexuality, defined as the desire for sex and satisfaction with sexual activity, has been found to be lower in IBD patients [41]. There are multiple causes for this finding including the disease itself, medications, surgery, and its influence on energy level, libido, mood, and body image. Indirectly, there may be symptoms of fatigue and poor body image following surgery and use of medications like corticosteroids. Marín and colleagues found that 50% of women and 33% of men reported worsening sexual function after the diagnosis of IBD [42]. The disease itself may lead to issues with symptoms of diarrhea, fear of fecal incontinence, flatulence, and fistula drainage [43]. Impaired sexual activity is particularly worse in women with IBD compared to men with IBD; women with IBD were more likely to have impaired body image and decreased libido and engage in less intercourse [44]. In another study, women with IBD had lower sexual activity compared to age matched controls, but, interestingly, partner satisfaction was equally high [41]. The major factor affecting sexual activity, however, was related to concomitant depressed mood rather than disease activity [45]. As a result, in patients with reduced sexual satisfaction, it is important to both assess disease related issues and also screen for other potential causes like depression.

#### 2.4.2. Sexual Issues after Surgery

Additional issues affecting sexual well-being are surgical treatments for IBD. Following surgical treatment for IBD, there is a significant concern of sexual dysfunction and dyspareunia [46]. Following a proctocolectomy in patients under 40, 33% of women complained of reduced quality of sexual life and 22% noted reduced satisfaction following intercourse after the surgery [47]. However, other studies have observed an improvement in sexual function. Metcalf et al. found that patients who undergo proctocolectomy with formation of either a Kock pouch or ileoanal anastomosis were more likely to increase their frequency of intercourse and had a reduction in the incidence of dyspareunia [48]. Damgaard et al. similarly reported finding increased sexual function and quality of life following ileal J-pouch anastomosis [49]. In a prospective study following ileal pouch-anal anastomosis, Davies et al. found that while abnormal sexual function decreased from 73% to 25% after surgery, the improvement in female sexual function took an average of 12 months postoperatively [50]. The type of surgical approach, whether laparoscopy versus conventional restorative proctocolectomy, did not lead to a difference in quality of life outcomes; however, laparoscopy is associated with improved cosmoses and body image which can affect one's sexual health [51]. In our center, our colorectal surgeons favor a laparoscopic approach whenever feasible.

Stoma formation carries many concerns with regard to its effect on sexual health. After formation of stoma, individuals have concerns about appliance leakage, odor, and body image [46]. Follick et al. surveyed patients with ostomies with 78% reporting a decrease in sexual activity; 34% had decreased enjoyment from sexual relations, and 41% felt sexual relations were a problem [52]. However, following ileostomy, patients noted unchanged or improved sexual function [53]. With adequate patient counseling regarding possible issues that can interfere with sexual relations and having adequate support services for the patient to address these issues if/when they come up, many of the concerns related to changes in sexual relations after surgery can be mitigated [54, 55]. As a result, we strongly recommend being proactive and initiating this discussion about sexual well-being with patients as a routine piece of the clinical assessment of women with IBD.

### 2.5. Fertility, Family Planning, and Contraception

#### 2.5.1. Fertility

Women with IBD are often reluctant to discuss issues of family planning with their gastroenterologists. Fear of the unknown including likelihood of pregnancy, disease activity, its effects on an unborn child, and heritability of IBD in offspring may interplay with one another. This is particularly evident in a 2009 survey of patients with IBD who were queried on their views of subjective and objective views about fertility. While 42% of patients described some fear of infertility, the rate of those seeking medical infertility advice was no different compared to the healthy population. Specific concerns identified in this population included IBD heritability, medication teratogenicity, and risk of congenital abnormalities [56]. In light of these concerns, multiple studies have unsurprisingly shown that voluntary childlessness is more common in women with IBD compared to healthy controls. In a study by Marri et al., 18% of CD and 14% of UC patients chose voluntary childlessness compared to 6% in healthy controls. Contraception use in the IBD cohort was lower than controls prior to the diagnosis of IBD but higher than healthy controls afterwards [57]. Similarly, a recent survey found that nearly 80% of the women with IBD who did not have children in the study had chosen not to have kids and did not have any fertility issues [58].

It is important to note though that fertility is in fact reduced in cases where patients undergo surgery and have an IPAA. A meta-analysis by Waljee et al. reported the risk of infertility was increased threefold [59]. The weighted average infertility rate was 48% after IPAA [59]. The exact cause of this reduction in fertility is unknown. It is speculated that either it is related to the surgical manipulation in the pelvic area or secondary to adhesions resulting in damage to the reproductive organs [60]. Therefore, while not an ideal surgery, some may consider either a temporary diverting ileostomy or temporary ileorectal anastomosis until after childbearing is completed to avoid this risk reduction. However, Pabby et al. found that women who had underwent IPAA were able to achieve live births following in vitro fertilization at rates comparable to women with UC without IPAA and women unaffected by IBD [61].

#### 2.5.2. Family Planning

It is thus important for the clinician to broach the possibility of pregnancy and importance of family planning. Ideally, women should have quiescent disease when conception occurs, as this portends a favorable prognosis for both mother and baby. The presence of active disease may cause lower rates of fertility, but, as mentioned above, when disease is in remission, fertility is similar to those without IBD [62, 63]. The risk of passing IBD on to an offspring ranges from 1.6 to 5.2% with one parent having the disease and up to 36% when two parents have the disease [64, 65]. Only two of the medications, methotrexate and thalidomide, used to treat IBD are category X agents and should absolutely not be used [63]. The majority of medications used to treat IBD are otherwise either category B or category C [63]. If the disease is in remission, the effect of IBD on pregnancy is generally thought to be minimal. Some studies have indicated risks of preterm birth, low birth weight, and small gestational age. However, all of these are more significant when the disease is active [60, 63]. Given the importance of staying on medication to treat the IBD and risk of complications if the disease is/becomes active, we recommend that all women with IBD who plan on conceiving be seen by a high-risk obstetrician and followed up closely by their gastroenterologist. A discussion regarding the management during pregnancy is beyond the scope of this review [63].

#### 2.5.3. Contraception

Specific considerations should be given when advising contraception use in women with IBD. While all forms of contraception are available to this population including barrier protection, oral contraception, and intrauterine devices, the optimal method of contraception should have a low failure rate and minimally interfere with IBD. Failure rates of barrier protection as well as case reports of IBD flares occurring after IUD insertion should be considered [66, 67]. Authors have also suggested that the risk of thrombosis in oral contraceptives (OCP) may theoretically exacerbate a known risk of thrombosis in IBD [68]. However, there have been no trials to date linking the use of OCPs and compounded thrombosis risk in IBD. In our center, we do not limit the use of OCP even in patients with inflammation. We recommend usage of whatever contraception is recommended as the ideal modality by the gynecologist.

The interaction between oral contraception and concomitant medical therapy for IBD should also be considered. Antibiotics are commonly used in IBD. It has long been advised by physicians that use of antibiotics in the setting of OCP decreases the efficacy of OCPs [69]. The American College of Gynecology importantly notes though that this has been based on anecdotal reports. According to their 2006 report, only rifampin has been shown to be associated with lower oral contraceptive steroid levels [70]. The only guidance for OCP use has been through small prospective trials, which note that OCP levels were seen to be stable [71–73]. Absorption of OCPs has not been shown to differ from healthy controls based on predominantly ileal or colonic disease [68], although some expert opinions still suggest that there may be potential for decreased efficacy in CD with small bowel resection and/or malabsorption [74–76]. If breakthrough bleeding occurs on long term antibiotic therapy, alternative contraceptive methods should be used.

There has been some concern that OCP use can increase the risk of developing IBD or cause an increase in flares. A meta-analysis in 2008 found that, after adjusting for smoking, exposure to OCPs increased the risk of IBD, in particular CD [77]. In a prospective cohort study examining the incidence of the development of IBD in the Nurse's Health Initiative, OCP use was associated with a small increase in the development of CD but not UC [78].

Because of evidence linking OCP use and risk of IBD, it has been postulated that OCPs may induce flares. In a literature review of the interaction between OCPs and IBD, Zapata et al. [68] found that the prevalence of relapse was not statistically significant in CD [79] or UC [80]. In a large prospective cohort of 331 women with CD, OCP use with progesterone only, low dose estrogen (30–35 mcg estrogen), and high dose estrogen (50 mcg estrogen) did not affect relapse rates [81]. More recently, in a 2014 article, Gawron et al. found that nearly 20% of women with IBD on OCPs reported improved GI symptoms related to menses while on OCPs [82]. As noted previously, there are case reports of IBD flares within IUD days to 24 months after placement, but larger studies and definite causality are lacking [66, 67].

### 2.6. Menopause

#### 2.6.1. Menopause

Scant literature pertains to the effect of IBD on menopause and vice versa. However, women diagnosed with IBD may be diagnosed after the onset of menopause and the influence of hormonal changes on disease behavior is not clear.

A handful of articles give some insight into IBD and the timing of menopause. A study by Lichtarowicz et al. surveyed 196 women with CD from Wales about menstruation cycles, age of onset of menopause, smoking, and use of oral contraceptives. Forty-eight of these women underwent physiologic menopause with the mean age of menopause onset between 46 and 47 in the IBD group compared to 49.6 years in healthy controls [83]. In contrast, Kane and Reddy did not find any difference in median age of menopause in patients with IBD compared to healthy controls [84]. Additionally, there was no difference in occurrence of flares prior to or following the onset of menopause [84].

#### 2.6.2. Hormone Replacement Therapy

Use of hormone replacement therapy (HRT) has been questioned since the Women's Health Initiative demonstrated an increase in risk of breast cancer, coronary artery disease, stroke, and venous thromboembolism [85]. HRT and its effect on IBD are unclear. Interestingly, Kane's study found a protective and dose dependent effect of HRT on disease activity in the postmenopausal state, with women with IBD on HRT 80% less likely to suffer from flares compared to those who were not on HRT [84].

More recently, use of HRT has been correlated with increased risk of development of UC. Khalili et al. prospectively followed up 108,844 women on HRT. The risk of development of UC was increased and further increased with duration of HRT use [86]. This correlation was not seen with development of CD. However, the correlation between HRT and flaring IBD remains unknown.

## 3. Conclusion

The health maintenance of patient with IBD has been increasingly emphasized. IBD affects women in many important ways. For the female patient diagnosed with IBD, gastroenterologists should be proactive with discussions about the course of the disease and its impact on menstruation, family planning, and menopause. In adolescence, careful proactive counseling regarding impact of IBD on QOL is critical. Physicians need to discuss with patients the effects both IBD and the medications used to treat IBD have on the onset of puberty and possible delays in onset of menarche. Additionally, women should be counseled about the potential increased risk in cervical cancer and means to reduce these risks with HPV vaccination and pap smears. The effect of IBD as well as the surgical treatments and the resultant impact on sexual relations is significant. While some of the issues may improve (e.g., ileostomy take-down), other issues like depression need to be sought out by the physician. Physicians should establish an open dialogue with patients early on regarding their sexual health and any issues related to it. While establishing this dialogue, careful attention to family planning is critical. Patients should be counseled about the use of OCPs and physicians should discuss how to optimize their disease status prior to attempting any pregnancy. Aside from patients with an IPAA, overall fertility should not be affected by IBD. Given potential complications associated with pregnancy we strongly encourage comanagement of these patients with a high-risk obstetrician. Overall, counseling the women with IBD about potential changes in puberty, menstruation, fertility, pregnancy, sexual health, and menopause is critical to optimizing their care. Such discussions allow the patient to develop realistic expectations and allay fears and concerns about the disease and its impact on important milestones that often go unaddressed. Given the knowledge deficit in the patient population, it is of utmost importance to not only present these topics, but guide women through any concerns and questions. Ultimately, future studies are still needed to improve the overall medical management and surgical management and to also clarify ways to improve the QOL in women with IBD.
